# Impact of the COVID-19 pandemic on surgical practice, training, and research in Nigeria

**DOI:** 10.11604/pamj.2021.39.59.23678

**Published:** 2021-05-21

**Authors:** Musliu Adetola Tolani, Lovely Fidelis, Nasir Oyelowo, Aisha Mustapha, Wasiu Olusola Adebayo, Chike John Okeke, Ikechukwuka Ifeanyichukwu Alioke, Khalifa Ibrahim Abdulsalam, Afeez Ajibade Aruna, Nkemdilim Oyetola Okonji, Uche Albert Okeke

**Affiliations:** 1 Department of Surgery, Ahmadu Bello University, Ahmadu Bello University Teaching Hospital, Zaria, Kaduna State, Nigeria,; 2 Department of Obstetrics and Gynaecology, Ahmadu Bello University, Ahmadu Bello University Teaching Hospital, Zaria, Kaduna State, Nigeria,; 3 Department of Surgery, Lagos University Teaching Hospital, Idi-Araba, Surulere, Lagos State, Nigeria,; 4 Department of Surgery, Federal Teaching Hospital, Gombe, Gombe State, Nigeria,; 5 Department of Surgery, Central Hospital Benin, Edo State Hospital Management Board, Benin City, Edo State, Nigeria,; 6 Department of Maxillofacial Surgery, Ahmadu Bello University Teaching Hospital, Zaria, Kaduna State, Nigeria

**Keywords:** COVID-19, impact, surgery, training, research, Nigeria

## Abstract

**Introduction:**

the rising rate of SARS-CoV-2 infections has caused perceptible strain on the global health system. Indeed, this disease is also a litmus test for the resilience of the structures in the African health system including surgery. Therefore, this study aimed to determine the impact of the COVID-19 pandemic on surgical practice, training and research in Nigeria.

**Methods:**

it was a cross-sectional study conducted over three weeks in Nigeria among doctors in 12 surgery-related specialties. Consenting participants filled a pre-tested online form consisting of 35 questions in 5 sections which assessed demographics, infection control measures, clinical practice, academic training, research program, and future trends. Data were analyzed using Statistical Package for Social Sciences Version 20.

**Results:**

a total of 384 respondents completed the form. Their mean age was 38.3 years. Lockdown measures were imposed in the state of practice of 89.0% of respondents. Most participants reported a decrease in patient volume in outpatient clinics (95.5%) and elective operations (95.8%) compared to reports for emergency operations (50.2%). They also noted a decrease in academic training [Bedside teaching (92.1%), seminar presentation (91.1%) and journal presentation (91.8%)] and research (80.5%). Except in bedside teaching, those who had other virtual academic programmes were thrice the number of those who used in-person mode for the events.

**Conclusion:**

COVID-19 pandemic has caused a significant change in pattern and a decrease in the volume of patients seen by surgeons in their practice as well as a decrease in the frequency of academic programs and research activities in Nigeria.

## Introduction

The abrupt spread of SARS-CoV-2 infection across international boundaries has created a lot of challenges for workers in the healthcare system. These are certainly trying times for surgeons who are faced with the battle of drawing a delicate balance between continuing patient care at normal capacity and scaling down activities to essential operations in the quest to maintain workplace safety amid little or no evidence of a very effective treatment for the disease [1,2]. As a precautionary measure to reduce infection spread in hospitals, it is advised that patients are considered SARS-CoV-2 positive by healthcare workers until proven otherwise [3]. This however demands the availability of personal protective equipments, personnel, and other infrastructures, which are also being advocated to be judiciously utilized due to global scarcity [4]. In some institutions, measures to reduce non-essential physical contact of doctors with patients have been implemented through the use of formal phone consultations and the harnessing of the opportunities in telemedicine [5]. Despite all these, the paranoia of getting infected or spreading the infection to family and close friends of healthcare workers could affect the making of a timely decision on different aspects of patient management [6].

The consequence of this change in the pattern of clinical care might resonate far beyond patient care as hands-on training, surgical drills, seminars and some research work have been practically suspended [7]. While virtual meetings have been employed by some departments for continuous academic contact between the mentor and the mentee, limitations exist to the use of this technology in surgery where practical supervision is of utmost importance. There are currently guidelines on surgical care and operation in confirmed or suspected cases of SARS-CoV-2 in different parts of the world [8-10]. However, it is said that the COVID-19 pandemic is a litmus test of the resilience of the healthcare system in all countries in sub-Saharan Africa. At present, its exact impact on various fields of application of surgery in Nigeria is not known. The aim of this study was to determine the impact of the COVID-19 pandemic on surgical practice, training and research in Nigeria.

## Methods

**Study design, population, and setting:** it was a cross-sectional study conducted prospectively over three weeks between 24^th^ April 2020, and 15^th^ May 2020. The study population included surgeons with various levels of professional experience in 12 surgery-related specialties working in federal, state, and private tertiary and secondary healthcare centers in any state in Nigeria. Doctors not working in surgery-related specialties, retired surgeons, and those not practicing in Nigeria were excluded from the study. Nigeria is a tropical country on the Gulf of Guinea in sub-Saharan Africa. It lies between latitudes 4°N to 14°N and longitudes 2°E to 15°E and has a total land area of 923,768 km^2^ [11]. The current population estimate of the country is 205,530,064 [12]. Nigeria has 36 states and the Federal Capital Territory, which are divided into six geopolitical zones. Operations of increasing complexity are offered in ranked tiers of her healthcare system in all states of the federation by trained surgeons, medical officers and residents, and interns undergoing training in recognized post-graduate medical colleges or teaching hospitals in the country. Asymptomatic SARS-CoV-2 cases, those with clinical suspicion or confirmed cases of SARS-CoV-2 could present to these healthcare professionals for the management of their surgical condition.

**Sample size calculation:** sample size was calculated using the Fischer´s formula [13]. Assumptions of a standard normal variate (z) of 1.96 at 5% type 1 error and an absolute error at precision (d) of 0.05 was made. In the setting of this study with an unknown prevalence of the impact of COVID-19 pandemic on surgical practice in Nigeria, this value (p) was assumed to be 50%. Thus, the sample size was computed as 384.

**Data collection:** data collection was done using a pre-tested online google form which had 35 questions in 5 sections. Close-ended questions, dichotomous response format, and a three-point Likert scale were used to assess these areas as appropriate. Information obtained included data on demographics (age, gender, state of practice, type of health institution, professional cadre, number of years of professional practice and surgical specialty), infection control practices (existence, type and duration of lockdown measures, history of operation on a patient with SARS-CoV-2, history of the use and the duration of obtaining a pre-operative molecular diagnosis for SARS-CoV-2, availability and source of personal protective equipments and the practice of surgical asepsis), dynamics in clinical practice (change in patient volume in various surgical service areas and change in the use of telemedicine for practice since the pandemic, psychological effect of the pandemic on clinical practice and the areas of patient management affected by this fear), changing aspects of academic training and research program (variation in the frequency of bedside teaching, seminars, journal clubs and research activities, the choice of in-person and (or) virtual mode for these activities, the extent of supervision of the training and the efficiency of virtual meetings) and future trends (likelihood of adopting virtual meetings, telemedicine and (or) telesurgery, electronic surgical booking and compulsory pre-operative SARS-CoV-2 test for surgical practice and training in the post-COVID period). The data obtained was converted into a Microsoft Excel Spreadsheet format and then opened on a statistical analysis software.

**Statistical analysis:** data were analyzed using Statistical Package for Social Sciences Version 20. The categorical variables were summarized as frequency and percentages while continuous variables were summarized as mean and standard deviation or median and interquartile range as appropriate. Some of these data were displayed using tables and charts.

**Ethical consideration:** ethical approval was obtained from the Ahmadu Bello University Teaching Hospital Health Research and Ethics Committee (ABUTHZ/HREC/W37/2020). Only consenting individuals were recruited into the study. Participation was voluntary and no record of participants´ identity was required while filling the questionnaire.

## Results

**Figure 1:**
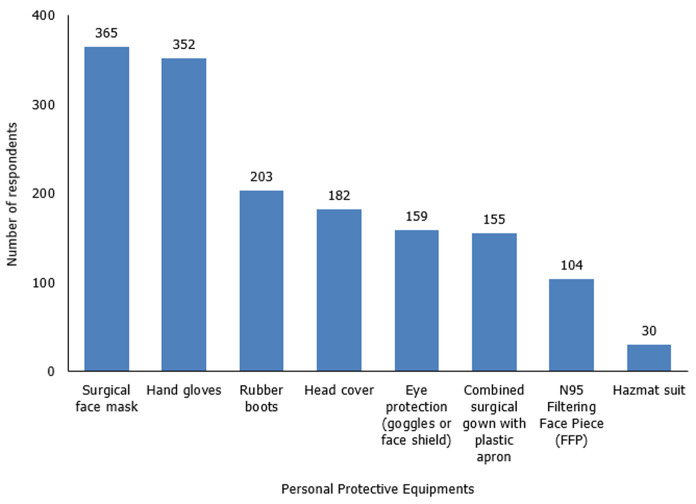
availability of personal protective equipments among respondents

A total of 384 responses were analyzed. The mean age of the respondents was 38.3 ± 7.0 years. Three hundred and fourteen (82.0%) were males while 69 (18.0%) were females with a male to female ratio of 4.6: 1.0. Over two-thirds of the participants, 267 (69.7%) worked in federal teaching hospitals. The median number of years of professional surgical practice of the respondents was 10.0 (5.0 - 12.0) years. The forms were submitted by participants from 34 states in Nigeria and the Federal Capital Territory. The distribution of the respondents across the six geopolitical zones in the country and across the 12-participating surgery-related specialties are shown in Table 1. As a result of COVID-19 pandemic, lockdown measures had been imposed in the state of practice of 341 (89.0%) of the respondents for an average duration of 4.0 (3.0 - 5.0) weeks. However, only 158 (41.3%) of these surgeons live in an area where total lockdown had been imposed. Additional measures to break intra-hospital transmission included the use of personal protective equipments. The majority of respondents attested to the availability of surgical face masks, 365 (95.1%), and hand gloves, 352 (91.6%). The account of respondents regarding accessibility to other components of personal protective equipment is shown in Figure 1. These equipments were mostly supplied by the respondents´ primary health institution, 304 (79.2%). However, 164 (42.7%), 37 (9.6%), and 22 (5.7%) obtained these items from personal sources, through other means, and from non-governmental organizations respectively. On the subject of pre-operative molecular screening test for SARS-CoV-2, ten of the respondents (2.6%) had carried it out for suspected SARS-CoV-2 cases in their practice. The median duration of obtaining the test result following the sample collection was 3.5 (2 - 5) days. However, only 1 (0.3%) of the respondents had operated on a SARS-CoV-2 positive patient during the study duration. The proportion of respondents who rated the practice of surgical asepsis high during the pandemic was 10% more than those that gave a similar rating before the pandemic (139 [36.8%] versus 103 [26.9%]).

**Table 1:** demographic characteristics of the respondents

Demographic characteristics	Frequency	Percentage
**Institution of practice**		
Federal teaching hospital	267	69.7
Federal medical center	43	11.2
State specialist hospital	24	6.3
State teaching hospital	23	6.0
Private hospital	15	3.9
State general hospital	11	2.9
**Geopolitical zone**		
North West	148	38.7
South West	64	16.8
North Central	60	15.7
North East	51	13.4
South East	37	9.7
South South	22	5.8
**Surgical specialties**		
Obstetrics and gynaecology	88	23.9
General surgery	64	17.4
Urology	42	11.4
Orthopaedic and trauma surgery	30	8.2
Otolaryngology	25	6.8
Oral and maxillofacial surgery	24	6.5
Neurosurgery	22	6.0
Cardiothoracic surgery	19	5.2
Ophthalmology	16	4.3
Paediatric surgery	14	3.8
Plastic surgery	13	3.5
Anaesthesia	11	3.0
**Number of years of professional practice**		
1 - 5 years	103	27.1
6 - 10 years	126	33.2
11 - 15 years	96	25.3
16 - 20 years	29	7.6
21 years and above	26	6.8

**Figure 2:**
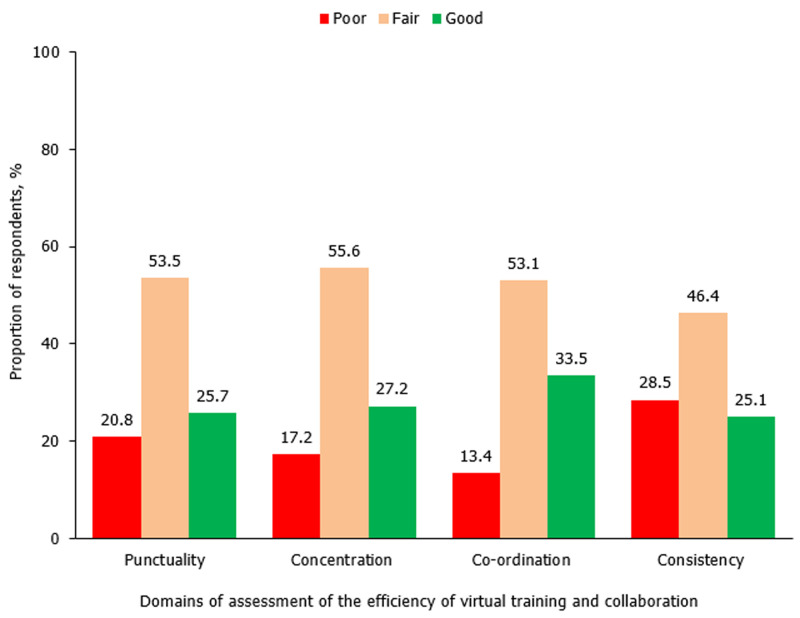
perceived efficiency of use of virtual training and online research collaboration in the academic programs

**Figure 3:**
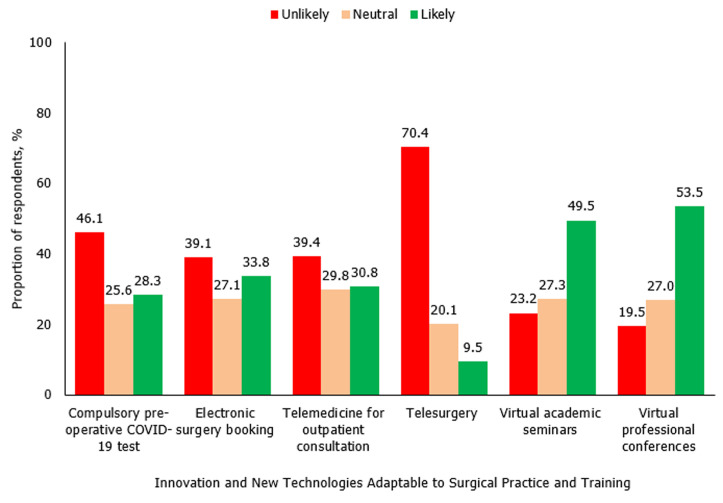
likelihood of adopting innovation and new technologies to surgical practice and training in the post COVID-19 era

The surgical practice of the majority of the respondents, 313 (81.5%) had been affected by the fear of being infected with the virus in the line of duty. Specifically, 280 (72.9%), 199 (51.8%), and 149 (38.8%) of the survey participants highlighted that performing clinical evaluation, carrying out peri-operative care, and making clinical decisions respectively had been adversely affected by this fear. A decrease in patient volume in various surgery service areas, in different educational programs for residency training and in the capacity for research was reported by the majority of the respondents as shown in Table 2. Regarding the mode of delivery of the academic program for surgical training, 142 (37.0%) of the respondents noted that bedside teaching occurred via physical contact while 122 (31.8%) of them observed that telemedicine was used for the teaching. On the other hand, the proportion of respondents who reported the use of telemedicine for seminar presentations (190 [49.5%] versus 61 [15.9%]), journal meetings (133 [34.6%] versus 48 [12.5%]) and research clubs (157 [40.9%) versus 50 [13.0%]) were about three times the percentage of those who used in-person approach for these academic events. An analysis of the efficiency of use of virtual training and online research collaboration showed that around half of the respondents rated it as fair in the domains of concentration (55.6%), co-ordination (53.1%), punctuality (53.5%) and consistency (46.4%) as shown in Figure 2. These virtual gatherings were the most likely of all future innovations and technologies that can be readily adapted to surgical training, research, or practice in the post-COVID-19 era in Nigeria as shown in Figure 3.

**Table 2:** perceived effect of SARS-CoV-2 pandemic on patient volume, academic training and research activities

Areas	Number of respondents, n (%)		
	Decreased	Unchanged	Increased
**Patient volume**			
Out-patient clinic	365 (95.5)	14 (3.7)	3 (0.8)
Ward admission	360 (94.0)	20 (5.2)	3 (0.8)
Clinic procedure	367 (96.3)	14 (3.7)	0 (0.0)
Elective operation	365 (95.8)	16 (4.2)	0 (0.0)
Emergency operation	191 (50.2)	161 (42.4)	28 (7.4)
**Academic training**			
Bedside teaching	348 (92.1)	28 (7.4)	2 (0.5)
Seminar presentation	347 (91.1)	26 (6.8)	8 (2.1)
Journal presentation	346 (91.8)	29 (7.7)	2 (0.5)
**Telemedicine utilization**	22 (6.1)	189 (52.5)	149 (41.4)
**Research capacity**	306 (80.5)	51 (13.4)	23 (6.1)

## Discussion

Since the first detected case of severe acute respiratory syndrome coronavirus 2 (SARS-CoV-2) infection in Nigeria on 27^th^ February 2020, the daily figures have risen to a peak of 386 confirmed infections in the second week of May 2020. At present, the country has 6,175 confirmed cases and 191 deaths from the virus pandemic [14]. The stretch in scarce human and material hospital resources imposed by this pandemic has resulted in sudden and unplanned changes in all aspects of surgical practice, especially in settings where both SARS-CoV-2 and non-SARS -CoV-2 positive patients are cared for [15]. To our knowledge, this is the first survey describing the spectrum of alteration in training, research, and practice of surgery in Nigeria due to the coronavirus pandemic. Respondents in this study were distributed across twelve surgical specialties. This might be because accredited training in these areas are locally available under the post-graduate medical colleges of Nigeria and West Africa. Their wide distribution in terms of years of professional practice affords this study the unique opportunity of gaining the perspective of young foot soldiers of the profession who are usually the first in the line of duty and their experienced trainers.

The surgeon is particularly at risk of getting the infection in the hospital environment, most especially the operating theatre, due to the higher risk of droplet spread from aerosol-generating procedures (endotracheal intubation, gastrointestinal endoscopy, airway suctioning, and tracheostomy) or contact with undiagnosed asymptomatic viral carriers during the 3 to 14 days incubation period [16,17]. This thus raises the question of the use of personal protective equipments by surgeons for the seamless but safe conduct of surgical activities. Basic personal protective equipment items like facemasks and hand gloves were available to almost all the respondents in this study. However, N95 filtering Facepiece and hazmat suit were only available to 27.1% and 7.8% of respondents. This is not unusual as there is a recognized scarcity of high-end personal protective equipments worldwide. However, it could mean that during the rational allocation of obtainable items, theatre suites and intensive care units should be considered as a priority due to the anticipation of emergency operations on suspected or confirmed cases [18]. In the same vein, the re-iteration of the importance of contact precautions including the reduction of operating room staff and prevention of inflow of personal belongings into the theatre is important and this could have contributed to the respondents´ attribution of a higher rate of practice of surgical asepsis during the pandemic [10,19]. There is however a need to improve the rate of pre-operative testing for SARS-CoV-2 in emergency cases above the low rate of 0.3% in respondents of this study. This might be a herculean task considering the poor proximity of some test centers to the surgeon as well as the long turn-around time (six to twenty-four hours) needed for the completion of a reverse transcriptase-polymerase chain reaction test for SARS-CoV-2 [20].

The high rate (81.5%) of fear of being infected with coronavirus by healthcare workers in this study is not surprising. Similar to this study, Consolo *et al*. in a survey among dental practitioners in Italy reported that 83.4% of them were afraid of being infected with the virus during their professional activity. This fear increases the level of psychological distress in these surgeons and could lead to lower work morale [21]. In order to prevent physical contact during various aspects of surgical practice, electronic medical records with audiovisual capabilities or alternative telehealth facilities such as telephones, skype calls, google meet, duo, facetime, and zoom meetings can be used [5]. Although almost all respondents (95.5%) reported a reduction in the volume of patients seen in the outpatient clinic in this present study, there was an increase in the rate of use of an alternative telemedicine approach in about 2 in 5 of them. The rate of uptake of this technology is similar to the figure of those who had a pre-visit by telephone [65 (41.7%)] reported by Maffia *et al*. in their survey [22]. The role of this technology as a screening tool for maintaining clinics in those with time-sensitive investigation reports or symptomatic patients requiring urgent review and intervention cannot be overemphasized [4].

Even though surgical expertise is not directly needed for the care of most patients with SARS-CoV-2 infection, our study showed a dramatic reduction in the number of elective operations during this pandemic. This could be related to a reduction in outpatient patient volume and hospital admissions, cancellation of booked cases, and limitation to the performance of only selected cases like cancer operations perhaps occasioned by local hospital policies, statewide lockdown measures, and fear of contagion by patients. Although this might be a stopgap measure for infection control, it could worsen the surgical disease burden and create a future strain on the weak healthcare system in Nigeria [19,23]. In contrast to the abrupt drop in patient volume in other surgical activities, only 50.2% of respondents reported a decrease in the frequency of emergency cases. The role of optimal and timely interventions in these acute conditions cannot be overemphasized [17]. Novara *et al*. in their study also observed that 50% of typical emergency consultations presented to the hospital during this pandemic. However, these cases received the specialist services required [24].

The development of a sound clinical knowledge base and unique skill set of a surgeon requires exposure to varying certified curricula-based activities. The pandemic has however compelled trainers and trainees to adapt to demands for a safe execution of educational activities by vacating the traditional face-to-face training in favour of remote learning and collaborations in seminars, journal clubs, and research meetings as shown in our study. This creates an ample opportunity to further develop communication skills and networking for research partnerships using the new platform. The figures in this study which showed similar rates of respondents who were involved in physical and virtual bedside teachings could however imply difficulty in adapting this new structure to clinical skill acquisition. However, innovative styles like need-based workflow restructuring for critical care and emergency surgery postings, virtual didactic sessions, and task training with video feedback could be introduced to ensure the continuity of surgical education and development of the required competencies [25].

## Conclusion

The advent of the COVID-19 pandemic has a great effect on the pattern of surgical practice and volume of patients seen by surgeons in Nigeria. It has also resulted in a decrease in the frequency of surgical academic programs and research activities. Technologies are being introduced to drive the continuation of training and research during this period. Some of these innovations have a bright prospect of being incorporated into these areas of surgery after the pandemic.

### What is known about this topic

- COVID-19 is a novel and ongoing pandemic;
- Its ravaging effect on surgery has been studied and documented mostly among the Asian and European populations, where there has been a high proportion of cases of surgical intervention on COVID-19 positive individuals.

### What this study adds

- The COVID-19 pandemic has caused a major reduction in the volume of patients seen in elective settings in surgical practice in Nigeria;
- Around four in five surgeons also reported a perceived decline in their research capacity during this period;
- The consideration for safe learning has however driven the introduction of innovation in surgical training with adaptation to a sandwich of virtual and face-to-face training.
